# Prenatal diagnosis and genetic etiology analysis of talipes equinovarus by chromosomal microarray analysis

**DOI:** 10.1186/s12920-023-01733-2

**Published:** 2023-11-20

**Authors:** Xiaorui Xie, Baojia Huang, Linjuan Su, Meiying Cai, Yuqin Chen, Xiaoqing Wu, Liangpu Xu

**Affiliations:** 1 Medical Genetic Diagnosis and Therapy Center, Fujian Key Laboratory for Prenatal Diagnosis and Birth Defect, Fujian Provincial Maternity and Children’s Hospital, No. 18 Daoshan Road, Gulou District, Fuzhou, 350001 China; 2 Prenatal Diagnosis Center, Quanzhou Maternity and Children’s Hospital, Quanzhou, China

**Keywords:** Talipes equinovarus, Karyotyping, Single nucleotide polymorphism array, Chromosome, Copy number variations

## Abstract

**Background:**

With the advancement of molecular technology, fetal talipes equinovarus (TE) is believed to be not only associated with chromosome aneuploidy, but also related to chromosomal microdeletion and microduplication. The study aimed to explore the molecular etiology of fetal TE and provide more information for the clinical screening and genetic counseling of TE by Chromosomal Microarray Analysis (CMA).

**Methods:**

This retrospectively study included 131 fetuses with TE identified by ultrasonography. Conventional karyotyping and SNP array analysis were performed for all the subjects. They were divided into isolated TE group (n = 55) and complex group (n = 76) according to structural anomalies.

**Results:**

Among the total of 131 fetuses, karyotype analysis found 12(9.2%) abnormal results, while SNP array found 27 (20.6%) cases. Trisomy 18 was detected most frequently among abnormal karyotypes. The detection rate of SNP array was significantly higher than that of traditional chromosome karyotype analysis (*P* < 0.05). SNP array detected 15 (11.5%) cases of submicroscopic abnormalities that karyotype analysis did not find. The most common CNV was the 22q11.2 microdeletion. For both analyses, the overall detection rates were significantly higher in the complex TE group than in the isolated TE group (karyotype: *P* < 0.05; SNP array: *P* < 0.05). The incremental yield of chromosomal abnormalities in fetuses with unilateral TE (22.0%) was higher than in fetuses with bilateral TE (19.8%), but this difference was not statistically significant (P > 0.05). Abnormal chromosomes were most frequently detected in fetuses with TE plus cardiovascular system abnormalities.

**Conclusion:**

Fetal TE is related to chromosomal microdeletion or microduplication. Prenatal diagnosis is recommended for fetuses with TE, and CMA testing is preferred. CMA can improve the detection rate of chromosomal abnormalities associated with fetal TE, especially in pregnancies with complex TE.

## Introduction

Talipes equinovarus (TE) manifested as rotational foot is a common distal limb deformity. It may affect the bones, connective tissue, muscles, vascular or nerve structures of the limbs. The incidence varies among different countries, with an average rate of nearly 1–3 per 1000 live births [1, 2]. The fetal foot deviates from the midline from the ankle, bends and turns inward, and is fixed in this position, and the posture does not change dynamically to rule out positional TE [3, 4]. TE can be unilateral or bilateral, isolated or accompanied by other malformations, and is generally treated with non-operative methods to gradually manipulate the foot to a corrected position, although some sever cases finally require surgical intervention [5, 6].

The aetiology of TE is complex and involves both genetic and environmental factors, including maternal and paternal smoking, maternal obesity, oligohydramnios, twin pregnancy and amniotic band syndrome [7, 8]. It has also been associated with restricted fetal activity in utero, such as more researches focused on genetic etiology of TE. Aneuploidies including trisomy 18, trisomy 21, trisomy 13, and 47,XXY have been reported in fetuses with TE [9–11]. With the widely application of chromosomal microarray analysis (CMA) in prenatal diagnosis, many karyotype-undetectable abnormalities including microdeletions, microduplications, and region of homozygosity (ROH) were revealed in fetuses with TE [11–14]. Several genes, including PITX1, TBX4, IGFBP3, RBM10, UTX and CHD, have been found to be related to TE [11, 15–18]. Although some studies have used whole exome sequencing (WES) on TE, they were mainly applied in postnatal individuals [19, 20], and few studies have reported its use in prenatal samples.

The present study presented our experience using conventional karyotyping and single nucleotide polymorphism array (SNP array) to investigate the genetic etiology of TE in prenatal diagnosis.

## Materials and methods

### Patients and samples

The study involved 131 pregnant women who were admitted to the Fujian Provincial Maternity and Children’s Hospital in China between January 2017 and February 2023. All fetuses had been diagnosed with talipes equinovarus via ultrasonographic screening. The average maternal age was 30.9 years, ranging from 20 to 42 years old; and the gestational age ranged from 13 to 31 weeks. The specimens consisted of four cases of chorionic villus sample obtained through chorionic villus sampling at 9–13 weeks of gestation, 107 cases of amniotic fluid sampled through amniocentesis between 18 and 24 weeks of gestation, and 20 cases of cord blood sampled through cordocentesis after 25 gestational weeks. Fetuses with isolated TE and TE accompanied by soft ultrasound markers or other ultrasound malformations were classified into isolated TE group and complex TE group. The clinicians given the advices based on the results of the ultrasound and prenatal diagnosis, as well as the overall condition of the fetuses. Whether the pregnancy was terminated depended on the patients’ wishes ultimately and pregnancy outcomes were followed up. The clinical characteristics of the fetuses were summarized in Table 1. Written informed consent was obtained from all patients, and the present study was approved by the Protection of Human Ethics Committee of Fujian Maternity and Child Health Hospital.

**Table 1:** The clinical characteristics of 131 fetuses with TE and the detection rates of chromosomal abnormalities in different groups

indicators		Value and proportion	Detection rate (%)		
			karyotyping		SNP-array
Demographic characteristics					
Average of maternal age		30.9			
Gestational age(weeks)		13^+^~31^+^			
Male		86 (65.6%)			
Female		45 (34.4%)			
Ultrasonography findings					
Bilateral		81 (61.8%)	7.4%	19.8%	
Unilateral	Left-sided	18 (13.7%)	12.0%	22.0%	
	Right-sided	32 (24.4%)			
Isolated TE		55(42.0%)	0%	3.6%	
Complex TE		76(58.0%)	15.8%	35.5%	
Total		131(100%)	9.2%	20.6%	

### Conventional karyotyping

Fetal chromosome karyotype analysis was performed according to our laboratory’s routine procedures, with resolution at the 320–400 band level. Giemsa banding (G-banding) karyotypes were analyzed and diagnosed in accordance with the International System for Human Cytogenomic Nomenclature (ISCN2020) [21].

### Chromosomal microarray analysis and interpretation of CMA results

Fetal DNA was extracted using the QIAGEN DNA mini kit (Qiagen, Valencia, CA, USA). The DNA samples were analyzed using the CytoScan 750 K gene chip detection platform (Affymetrix Inc., CA, USA). CNV thresholds were set to report deletions greater than 200 kb or duplications greater than 500 kb. The data were analyzed using the Chromosome Analysis Suite (ChAS) software (Affymetrix, Santa Clara, CA, USA) and genomic imbalances were annotated based on the GRCh37/hg19 Genome Build (July 2013). SNP-array results were evaluated with reference to the following databases: Database of Genomic Variants (DGV, http://projects.tcag.ca/variation/), Database of Chromosomal Imbalance and Phenotype in Humans Using Ensembl Resources (DECIPHER, htts://decinher.sanger.ac.uk/), Consortium, and Online Mendelian Inheritance in Man (OMIM, http://www.omim.org/), the ClinGen database (https://www.clinicalgenome.org/) and the ClinVar database (https://www.ncbi.nlm.nih.gov/clinvar/). When appropriate, peripheral blood was extracted from the parents of the fetuses for SNP array detection. The nature of CNVs was determined according to standards and guidelines of the American Society of Medical Genetics (ACMG) for the interpretation and reporting of genetic CNVs. CNVs were classified into five levels: pathogenic, likely pathogenic, benign, likely benign, and variants of unknown significance (VOUS) [22]. The clinical significance of copy number variations (CNVs) was systematically evaluated by referring to the above-mentioned databases, scientific literature, and ultrasonography findings.

### Statistical analyses

Data were expressed as frequencies and rates. Statistical comparisons were performed using the Chi squared test, and *p* < 0.05 was considered statistically significant.

## Results

### Results of karyotype analysis

Out of the 131 cases, 119(90.8%) had a normal karyotype. Among the 12(9.2%) cases with abnormal karyotype, trisomy 18 was the most frequently detected abnormality, accounting for 5 cases (3.8%). Other abnormal karyotypes included 3 cases of trisomy 21, 1 case of trisomy 13, 1 case of chimeric sex chromosome abnormality (47,XXY), 1 case containing marker chromosomes, and 1 case of chromosomal structural abnormalities (47,XX,+i(18)(p10)).

### Results of CMA

CMA revealed normal SNP array results in 104 cases and abnormal results in 27 (20.6%, 27/131) cases. The detection rate of abnormalities was significantly higher than that of traditional chromosome karyotype analysis (9.2% vs. 20.6%, *P* < 0.05). Among the 27 abnormal results, 10 cases had numerical abnormalities, and 17 cases had structural abnormalities in chromosomes. In the 17 cases, one had large segments of duplication, and 15 had submicroscopic abnormalities that karyotype analysis did not find. Moreover, the source of the marker chromosome was confirmed to be the p15.33p11 region of chromosome 5 via SNP array. After searching the database and literature, we concluded that 8 of the submicroscopic abnormalities were pathogenic mutations, 8(one case had three ROH) were mutations with unknown clinical significance, and one with microduplications in 8q24.22q24.3 was likely benign. Details of the 14 cases with CNVs and 3 cases with LOH are presented in Tables 2 and 3. The most common CNV was the 22q11.2 microdeletion (n = 3), which could lead to the DiGeorge syndrome (DGS).

**Table 2:** Detection results and ultrasonography findings of 14 fetuses with CNVs

Cases	Maternal age	Karyotype result	SNP result	Size	Ultrasound findings	Clinical significance	Pregnancy outcomes
Variants of pathogenic or likely pathogenic							
1	25	46,XY	arr[GRCh37]15q11.2(22,770,422–23,082,231)×1	312Kb	Isolated unilateral TE	Pathogenic	Continued gestation
2	38	46,XY	arr[GRCh37]22q11.21(18,631,365–21,464,764) ×1	1.74 Mb	Isolated unilateral TE	Pathogenic	TOP
3	28	47,XX,+mar	arr[GRCh37]5p15.33p11(1,135,760–46,242,541) ×3	46.1 Mb	Bilateral TE + decreased FL/BPD and FL/HC ratios; small bust size; increased cardiothoracic ratio; cardiac axis to the left; inner diameter of the left and right pulmonary artery branches was narrowed; mild tricuspid and pulmonary valve regurgitation; increased echo of parenchyma of both kidneys; intestinal hyperechogenicity	Pathogenic	TOP
4	24	46,XY	arr[GRCh37]7q11.23(72,608,900–74,242,167) ×3	1.6 Mb	Bilateral TE + mild tricuspid regurgitation	Pathogenic	TOP
5	28	46,XY	arr[GRCh37]16p11.2(29,591,326–30,176,508) ×1	585 kb	Unilateral TE + absence of bilateral kidneys; oligoamnios	Pathogenic	TOP
6	30	46,XY	arr[GRCh37]17p12p11.2(15,759,453–20,547,625) ×3	4.7 Mb	Unilateral TE + variation of blood vessels; right subclavian artery vagal	Pathogenic	Live birth; normal physical development; nonverbal; hyperactive.
7	27	46,XY	arr[GRCh37]17p13.3p13.2(525-5204373) ×1	5.2 Mb	Bilateral TE + ventriculomegaly; cerebellar vermis dysplasia; smaller magenblase; hydramnios	Pathogenic	TOP
8	28	47,XX,+i(18)(p10)	arr[GRCh37]18p11.32p11.21(136227-15143715)×4	15.0 Mb	Bilateral TE + ECD; right subclavian artery vagal	Pathogenic	TOP
9	39	46,XY	arr[GRCh37]22q11.21(20,730,143–21,800,471) ×1	1.0 Mb	Bilateral TE + CPC; renal pelvis cysts; hydramnios	Pathogenic	TOP
10	38	46,XY	arr[GRCh37]22q11.21(18,636,749–21,464,764)x1	2.8 Mb	Unilateral TE + increased echo of ventricular	Pathogenic	TOP
Variants of benign or likely benign							
11	34	46,XY	arr[GRCh37]8q24.22q24.3(135,106,599–140,610,869) ×3	5.5 Mb	Bilateral TE + abnormal hand posture; increased NF thickness,	Likely benign	TOP
Variants of unknown significance							
12	31	46,XX	arr[GRCh37]8q24.11(118,225,220–118,884,779)x3	659.5Kb	Bilateral TE + micromandible	VOUS	TOP
13	27	46,XY	arr[GRCh37]13q14.3(52,649,105–53,172,866) ×3	524Kb	Bilateral TE + mild tricuspid regurgitation; slight separation of bilateral renal collecting system	VOUS	TOP
14	28	46,XY	arr[GRCh37]Xq28(154,748,825–154,828,315)x0	79.4Kb	Bilateral TE + separation of bilateral renal pelvis	VOUS	Live birth; bilateral TE

TOP: termination of pregnancy; FL: femur length; BPD: biparietal diameter; HC: head circumference; NF: nuchal fold; NT: nuchal translucency; ECD: endocardial cushion defect; CPC: choroid plexus cysts; CoA: coarctation of the aorta; SUA: single umbilical artery; VSD: ventricular septal defect; AC: abdomen circumference; SD: standard deviations; HL: humerus length; PLSVC: persistent left superior vena cava; CSP: cavity of septum pellucidum;

**Table 3:** Detection results and ultrasonography findings of 3 cases with LOH

Cases	Maternal age	SNP result	Size	Ultrasound findings	genes involved in AR diseases	imprinting genes involved	Clinical significance	Pregnancy outcomes
15	29	arr[GRCh37]3q13.13q21.2(107,945,077–125,298,512)x2 hmz	17.3 Mb	Bilateral TE + patent ductus arteriosus	ATP6V1A (607,027)/HGD (607,474)/IQCB1 (609,237)	None	VOUS, increased risk of AR diseases	Live birth; bilateral TE, mental/physical retardation
16	33	arr[GRCh37]3p26.2p25.1(2,886,527–13,828,221)×2hmz, arr[GRCh37]4p16.3p15.33(3,473,602–14,373,371)×2hmz, 5p13.3p11(31,554,333–46,313,469)×2hmz	10.9 Mb, 10.9 Mb, 14.8 Mb	Unilateral TE + pulmonary valve thickens and the echo widens; mild tricuspid and pulmonary valve regurgitation	TRNT1(612,907)/CRBN(609,262), DOK7(610,285)/LRPAP1(104,225), TARS1(187,790)/SLC45A2(606,202)	None	VOUS, increased risk of AR diseases	Live birth; right-sided TE
17	29	arr[GRCh37]6p22.3p21.31(24,654,265–35,934,695)x2 hmz	11.2 Mb	Bilateral TE + abnormal wrist joint; separation of bilateral renal pelvis	PSMB8 (177,046), MSH5 (603,382), VARS (192,150)	Unclear	VOUS, increased risk of AR diseases	TOP

### Distribution of chromosomal abnormalities in different groups

**Fig. 1:**
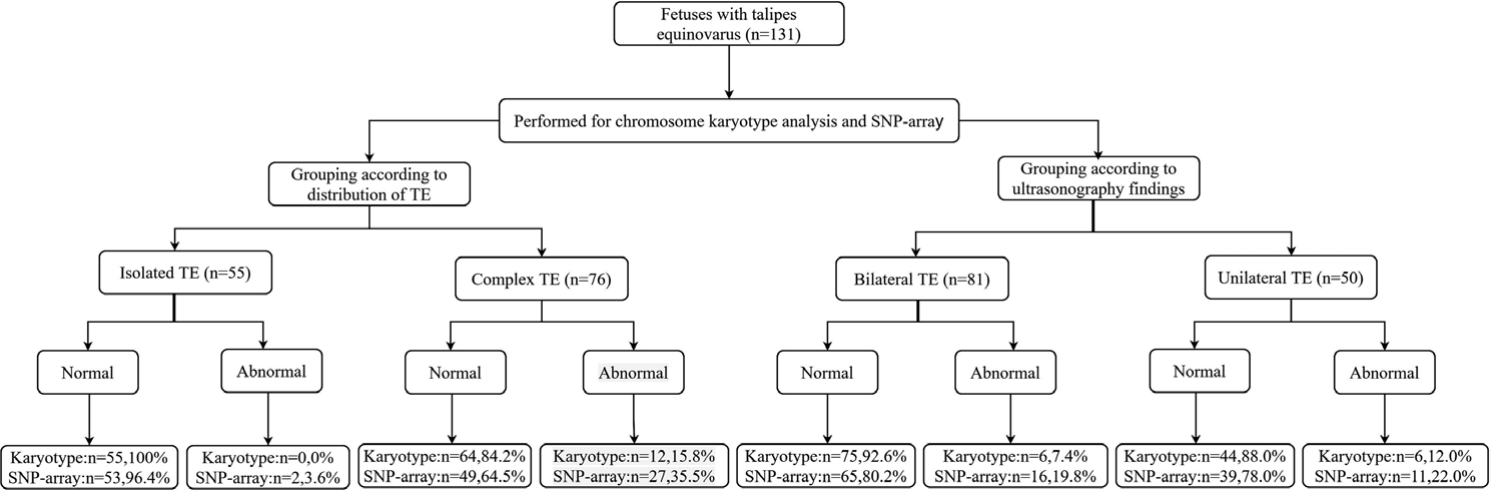
The flow chart of the chromosome detection

The detection rates of karyotype analysis were 0% (0/55) in the isolated TE group and 15.8% (12/76) in the complex TE group; the respective detection rates of SNP array were 3.6% (2/55) and 35.5% (27/76) (Table 1; Fig. 1). For both analyses, detection rates were significantly higher in the complex TE group (karyotype: 0% vs. 15.8%, *P* < 0.05; SNP array: 3.6% vs. 35.5%, *P* < 0.05).

Among the 131 fetuses with TE, 81 cases had bilateral TE and 50 cases had unilateral TE. The incremental yield of chromosomal abnormalities in fetuses with unilateral TE (22.0%) was higher than that in fetuses with bilateral TE (19.8%), but this difference was not statistically significant (*P* > 0.05).

### Distribution of concomitant ultrasonic abnormalities in cases with abnormal chromosomes

Among fetuses with complex TE, chromosomal abnormalities were most common in those with TE plus cardiovascular system abnormalities(n = 14), followed by those with TE plus urinary system(n = 7) and nervous system(n = 6) abnormalities. Table 4 shows the distribution of chromosomal abnormalities in fetuses with TE and different types of ultrasonic abnormalities.

**Table 4:** Distribution of chromosomal abnormalities in TE accompanied different body systems of associated structural abnormalities

The category of ultrasound abnormalities in other systems		numbers
Cardiovascular system	14	
Urinary system	7	
Nervous system	6	
Digestive system	3	
Respiratory system	1	
Facial abnormalities	6	
Other limb abnormalities	6	
Other ultrasonic anomalies*	12	

*This group was including the manifestations of polyhydramnios, oligohydramnios, increased NT or NF thickness, fetal growth restriction(FGR), fetal intrauterine growth retardation(IUGR), SUA and so on

### Pregnancy outcomes

Out of the 131 fetuses, 5 were lost to follow-up. The pregnancy outcomes of all cases were summarized in Table 5.

**Table 5:** Pregnancy outcomes of the total cohort

Pregnancy outcomes	Cases with normal chromosomes		Cases with chromosomal aberrations						
		Trisomy 21		Trisomy 18	Trisomy 13	Sex chromosome aneuploidy	Variants of pathogenic or likely pathogenic	Variants of benign or likely benign	Variants of unknown significance
Live birth with normal phenotype	72	0		0	0	0	0	0	0
Live birth with abnormal phenotype	6^a^	0		0	0	0	1^c^	0	3^e^
Termination of pregnancy	18^b^	3		5	1	1	8	1	3^f^
Continuation of pregnancy	3	0		0	0	0	1^d^	0	0
Lost to follow-up	5	0		0	0	0	0	0	0
Total	104	3		5	1	1	10	1	6

a: 6 cases were all with TE only; b: 16 cases underwent induced abortion due to severe malformation, and two cases resulted in miscarriage; c: Case 6 in Table 2; d: case 1 in Table 2; e: Case14 in Table 2, case 15 and 16 in Table 3; f: Case12 and 13 in Table 2, case 17 in Table 3

## Discussion

At around 11 weeks of gestation, the fetal lower limbs appear in their final physiological position. Talipes equinovarus(TE) can be diagnosed through ultrasonography from the late first trimester of pregnancy. Considering that some cases of TE may have delayed onset or be transient, the general diagnosis of gestational age is between 18 and 24 weeks [23, 24]. In our study, the gestational age range was between 13 and 31 weeks, including some cases diagnosed earlier and some cases referred late, as our center is a tertiary referral center. The incidence of TE varies by gender, with a ratio of males to females of about 2:1 [23]. The proportion (1.9:1) in this study was similar.

Chromosomal microarray analysis (CMA) is a powerful method to understand the pathogenesis of disease and provide more information for the evaluation of prognosis. CMA is a high-resolution technology for whole-genome analysis, detecting micro-deletions, micro-duplications, which are not routinely detected via karyotyping. CMA can be separated into comparative genomic hybridization (CGH) chips and SNP chips. The SNP array can detect ROH, triploidy, and maternal cell contamination, which the CGH array cannot [25]. In our study, the chromosome abnormality detection rate of karyotype analysis and SNP array analysis were 9.2% (12/131) and 20.6% (27/131), respectively, and the different detection rates were statistically significant. This finding was consistent with previous studies [12]. SNP array identified an additional 16 cases carrying submicroscopic abnormalities that escaped karyotype analysis and confirmed the source of a marker chromosome. Therefore, SNP array can complement the deficiency of traditional chromosome karyotype analysis and improve the detection rate of chromosome abnormalities in fetuses with TE (12.2%, 16/131). Moreover, our study found three cases with ROH, which contained the genes involved in autosomal recessive (AR) inherited diseases. It suggested an increased risk of AR inherited diseases. There was no clear imprinting gene in the ROH of case 1 and case 2. It has been reported that paternal ROH of chromosome 6 has been detected in about 20% of patients with Diabetes mellitus transient diabetes 1. The clinical phenotypes include fetal growth retardation (FGR), hyperglycemia, dehydration, macroglossia, etc [26]. Maternal ROH of chromosome 6 has also been reported in fetuses with FGR [27]. Whether the region of homozygosity in case 3 carries imprinting genes was unclear. The clinical significances of the three ROH were unknown. However, SNP-array provided more information about the genetic etiology and prognosis of fetuses with TE.

In the SNP array results, there was a high incidence of 22q11.2 microdeletion syndrome (DiGeorge syndrome, DGS) in fetuses with TE, which accounted for 17.6% (3/17) of all CNVs. Previous studies had shown that the incidence of fetal TE in DGS ranges from 1.1 to 13.3% [28, 29]. 22q11.2 microdeletion syndrome leads to the deletion of the *TBX1* gene. Some studies suggest that the *BTX4* pathway is highly related to the development of lower limbs and the pathogenesis of TE [30, 31], and it is speculated that the *TBX* family may play a role in the process. Therefore, the possibility of DGS should be considered when the fetus with TE presents with cardiovascular system abnormalities. In our study, the families of 3 fetuses with DGS chose to terminate the pregnancy, one of which was complicated with cardiac ultrasound abnormalities. In previous literatures, there were some cases of fetuses with DGS with isolated TE detected by CMA [12, 14]. It has been reported that the incidence of abnormal neurodevelopmental outcome was about 7% in fetuses with isolated TE [32]. And we detected several CNVs associated with abnormal development of the neuropsychiatric and skeletal musculoskeletal system using SNP array analysis. Specifically, case 7 showed a 659.5 kb duplication in the 8q24.11 region, which contained a partial fragment of the *EXT1*(608,177) gene associated with Multiple Exostoses type1. The clinical manifestations include short stature, pelvic exostoses, knee valgus and forearm deformity. In case 11, a microdeletion in p11.2 region of chromosome 16 was observed, which was 585 kb in size and contains 20 OMIM genes, including *TBX6* (602,427). This deletion can result in various clinical manifestations, primarily characterized by developmental delay, learning disabilities, speech disorders, autism spectrum disorders, bone dysplasia, mild facial abnormalities, cardiac malformation, epilepsy, and other symptoms. Case 9 carried a 4.7 Mb duplication in 17p12p11.2, which contain the *RAI1* gene that encodes the retinoic acid-inducing gene protein 1(*RIG-I*), and the mutation can cause Potocki-Lupski syndrome (17p11.2 duplication syndrome). The primary clinical features of the patients, included intelligence, mental retardation, short stature, autism, hyperactivity, high zygomatic arch, frontal eminence, palatal dysplasia, and cardiac dysplasia. Finally, case 14 showed a 15.0 Mb duplication that contained 57 OMIM genes in 18p11.32p11.21. Duplication of this region can lead to 18p tetrasomy, in which patients primarily present with intrauterine growth delay or restriction, feeding difficulties in infancy, microcephaly, intellectual impairment, finger/toe deformities, cardiac dysplasia, scoliosis, and hypotonia. While these CNVs are associated with the development of the neuromuscular system and skeleton, further research is required to determine whether they are direct causes of fetal TE.

Our study provides a comprehensive comparison of isolated and complex TE, as well as unilateral and bilateral TE. We found a significant difference in the overall detection rate between isolated TE and complex TE (2/55, 3.6% vs. 27/76, 35.5%, *P* < 0.05), which is consistent with previous studies [12, 14]. While interventional prenatal diagnosis of TE accompanied by other ultrasound anomalies is generally considered necessary during the prenatal period, the prenatal diagnosis of isolated TE remains controversial. Previous studies have shown that fetuses with isolated TE have a lower risk of chromosomal abnormalities [32–34]. However, some studies with different views have reported that ultrasound cannot screen all the abnormalities of fetuses, and some syndromes with chromosomal abnormalities have been found in fetuses with TE. In our cohort, we found that two fetuses with isolated TE carried the pathogenic CNVs, suggesting that isolated TE may indicate microdeletion or microduplication of chromosome. Additionally, the detection rate of chromosomal abnormalities in fetuses with unilateral TE was higher than that in fetuses with bilateral TE, but this difference was not statistically significant (11/50, 22.0% vs. 16/81,19.8%, *P* > 0.05). Based on our date, we recommended ultrasound reexamination when isolated TE is detected in a fetus. If ultrasound still shows fetal TE, whether unilateral or bilateral, prenatal diagnosis is recommended, while those who are negative should undergo routine prenatal examination. Prenatal diagnosis is recommended when ultrasonography indicates a fetus with complex TE, and CMA is preferred.

Previous studies have shown that nervous system abnormalities, urinary system abnormalities, cardiovascular system abnormalities and skeletal system abnormalities are the most common ultrasonic structural abnormalities associated with fetal TE [34–36]. Our study found that chromosomal abnormalities are most common in fetuses with TE and cardiovascular system abnormalities, followed by fetuses with TE and urinary system and nervous system abnormalities. Therefore, we believe that further examination of the fetuses with TE, such as magnetic resonance imaging (MRI), can improve the detection rate of other malformations, avoid missed diagnosis, and better assess the prognosis.

During the follow-up of cases, the majority of parents whose fetuses were detected with pathogenic chromosomal abnormalities chose to terminate the pregnancy. Additionally, there were 16 cases of fetal termination due to multiple malformations. Isolated TE can usually be treated and corrected by non-surgical means. The prognosis of such fetuses is generally good if there are no other malformations or genetic abnormalities. Therefore, we believe that in most cases, the pregnancy outcome largely depends on whether prenatal ultrasound combined with severe malformations and the chromosomal results of prenatal diagnosis.

There are some limitations to this study. Firstly, this study is retrospective, and the date lacks of some crucial parameters, such as family history, parental examination, and screening results of other skeletal and muscle diseases. At the same time, fetal TE may be related to a single gene or methylation [15–17, 19], and it’s regretful that we had not performed further tests such as whole exome sequencing (WES) in this study.

## Conclusion

Talipes equinovarus is a common congenital birth defect. CMA has been shown to increase the detection rate of chromosome abnormalities in fetuses with TE compared to karyotype analysis. This suggests that fetal TE may also be related to chromosome microdeletion or microduplication. Additionally, compared with cases of isolated TE, it is more recommended that fetuses with complex TE perform SNP-array analysis to confirm whether fetuses have chromosomal abnormalities. Upon the ultrasound diagnosis of TE, a comprehensive and detailed fetal screening, particularly of the cardiovascular system, should be performed. If the ultrasound reexamination result still shows TE, prenatal diagnosis is recommended, and CMA testing is preferred. Laboratories with the necessary resources can conduct further genetic testing to facilitate the prenatal diagnosis, consultation, and prognosis judgment for fetal TE.

## Data Availability

The datasets generated and/or analysed during the current study are available in the GEO repository, accession number GSE230532, https://www.ncbi.nlm.nih.gov/geo/query/acc.cgi?acc=GSE230532.

## Abbreviations

TE: talipes equinovarus
CMA: chromosomal microarray analysis
ROH: region of homozygosity
WES: whole exome sequencing
SNP: single nucleotide polymorphism
VOUS: variants of unknown significance
CNV: copy number variation
TOP: termination of pregnancy
FL: femur length
BPD: biparietal diameter
HC: head circumference
NF: nuchal fold
NT: nuchal translucency
ECD: endocardial cushion defect
CPC: choroid plexus cysts
CoA: coarctation of the aorta
SUA: single umbilical artery
VSD: ventricular septal defect
AC: abdomen circumference
SD: standard deviations
HL: humerus length
PLSVC: persistent left superior vena cava
CSP: cavity of septum pellucidum
DGS: DiGeorge syndrome
MRI: magnetic resonance imaging
CGH: comparative genomic hybridization
AR: autosomal recessive
